# The Modern Standard Business Honor1Abstract of an address delivered to the graduated classes at the University of Buffalo, May 31, 1907.

**Published:** 1907-08

**Authors:** Jeremiah W. Jenks

**Affiliations:** Ithaca. N. Y.; Professor of Political Economy and Politics at Cornell University


### The Modern Standard Business Honor.[1]

By JEREMIAH W. JENKS, A.M., Ph.D., Ithaca, N. Y.
Professor of Political Economy and Politics at Cornell University.

AT the outset, Professor Jenks said he did not intend to play the role of a destructive critic. He said his purpose primarily was not to defend, not to condemn either men or practices; but as best he might, to understand them. He commended President Roosevelt for the emphasis he laid upon the fact "that beyond and above questions of policy and governmental control was the question of personal honesty on the part of business-men and legislators—a fact too often lost sight of in our training to create professional skill. In college athletics we train men to win, often losing sight of the fact that victory by unfair means is disgrace, not honor. But I am not to try to fix your standards; but to analyze ways in which standards are fixed, and warn you not to drift, but to think."

Continuing, Professor Jenks said, in part:

"In spite of the pessimistic tone of many of our popular writers, it is certain that, in most fields of endeavor, society is improving in its methods and in its purposes. Why not in business efforts and business aims? The fact that within the last few years so many influential business men, not only in the United States but also in other countries, have engaged in operations which have shocked the sense of justice and honor and fair deal-

1. Abstract of an address delivered to the graduated classes at the University of Buffalo, May 31, 1907.

-ing, seems out of harmony with the general trend of social events. It was certainly not to be expected that men who seem entirely conscientious in all their dealings, public and private; men who have won universal respect, should suddenly in their business be found engaged in acts illegal and dishonorable.

"In explanation there seems only one alternative: Either our standard of business integrity has changed or we have found difficulty in applying our former standards to modern conditions. Doubtless within the last decade in business, there have been weak men in positions of importance who have yielded to temptation. But many acts condemned by public opinion have been committed by men who are not weak and who apparently have had no sense of guilt. No, the fact that there are weak or wicked men in business, while true, is not sufficient as an explanation.

### WHY MEN ARE CROOKED.

"We are all creatures of custom; we generally drift under the inertia of habit, in thought as well as in action. In consequence, changes in social or business conditions take us unawares; we need time to readjust ourselves. Meanwhile, we do foolish things, things that seem wrong. The old ways do not fit the new surroundings. This is a social fact never to be ignored. How does this principle fit our problem? What are the new conditions of business which may affect our standards of honor?"

The speaker declared that four characteristics of modern business had an important bearing upon this problem of business honor. First, business is done on a larger scale than ever before; secondly, the modern business man does not come into personal contact with his workingmen, his customers or the consumers of his products as did his predecessor of half a century ago; thirdly, there are corporations with directors and officers acting as trustees for thousands of stockholders; and fourthly, large profits from monopolies that are legal, but sometimes economically and socially unjustifiable, are more frequent now than earlier.

### THE EMPLOYER'S ALOOFNESS.

Professor Jenks then illustrated the principle of monopoly, showing that in a village or in a nation the same principle was operative. Passing to the second characteristic of modern business, the aloofness of the employer, Dr. Jenks declared that this removal from personal contact with laborer or customer "takes from business men the sense of cruelty and meanness which their acts would sometimes seem to have, if the consequences were immediate and personal. The rich man knows that he has so many hundreds of thousands of dollars invested in this or that corporation, but so long as he draws his dividends promptly, he is often content not to know more."

After detailing at some length the multifarious duties of the trustee or director of a railroad or a corporation, with the interests of many thousands of stockholders in his hands, Professor Jenks summed up that part of his argument as follows:

"But let us not be unjust. These excuses for wrongdoing are not confined to our great capitalists. It permeates our business life. We are like to magnify our offices. And we all in our business relation are very likely to employ similar methods in a small way. Look through the hundreds of columns of advertising matter in our daily press and in our magazines. Everywhere exaggeration of excellencies, concealment of defects, seldom bare, simple truth. Shall we conclude that most of us have a share in the guilt of our unscrupulous business magnates? I have been trying to see clearly the explanation of the evil practices in business life which have been so bitterly condemned. I have no excuses. I, too, condemn. But I am trying to understand. We must understand before we can intelligently seek the remedy."

WHAT IS THE CURE?

"What," asked the lecturer, "is the right standard of business honor? The aim of the state, i. e., of society organised for positive action, is to secure for its citizens not merely life, but abundant life of the highest type; economic prosperity with moral excellence. It is for us as citizens in a popular form of government to see how this can be best secured, and we must not consider only the distant ideals toward which we are pressing, but we must recognise with clear eye and patient heart the weakness of human nature and the conditions which place barriers in our pathway. Then only we must act.

"The duty, then, is imposed upon the government, upon us, of changing the conditions of business so that the ethical standards of our private life may be more promptly extended to our ever-changing business life. The state, for example, must in actual practice forbid the use of any means which are injurious to the public interest, such as the building of unsanitary factories or the employment of child labor. It must cut off the possibility of special favors to the strong; it must search out and detect dishonest practices of those whose wealth comes not from service, but from plunder and fraud, whether legal or illegal.

"Secondly, it is the duty of all of us to recognise that in business as well as in politics, the laborer is worthy of his hire. Each of us should feel that our work is primarily for the service of society; but we should also feel that when others are rendering a service to us or to society, they should receive the just reward of that service. The demand that a man whose services are actually worth $50,000 a year shall receive only $5,000, is as wrong economically and morally as that a skilled laborer shall receive only a pittance to keep him from starvation.

"Thirdly, the beneficial result of honesty, good laws and good service can best be secured by publicity. This must be attained in part by the sanction of the state, in part by the education of public sentiment. The chief reason why it is necessary to have business secrets is that the buying public is not willing to give the higher profit to the man who renders the better social service."

DAWN OF REFORM.

Professor Jenks concluded with an eloquent peroration on the future standards of business and political morality as presaged by the reforms of the last few years. He said in part:

"It is easy to see that the public moral sentiment of the world is already aroused. And we have every reason to believe that we shall see an ever accelerating progress. It is right for us to find faut with our politicians and our business men. They have deserved it, but we should also find fault with ourselves. Their crimes and cruelties are no more ignoble than our petty deceit and meanness, our selfishness and our concealments, our search for bargains, our eagerness to get on. The misdeeds had to be on a grand scale to attract our attention. But now we see them and see them clearly, we shall soon see farther. Hithertofore our business standard has really been the greatest possible reward for self in return for the least service. When we once see ourselves clearly, we shall see that the fitting standard is the greatest service possible for a just reward.

"It may seem a lame and impotent conclusion that there is no legislative panacea for our business ills, that upon us as individuals largely rests the responsibility for our improvement, and that the fundamental principle is, that clear-sighted and impartial observation of facts, including human nature, is our only safe guide in social reforms; that our only methods are the commonplace ones of preventing abuses and securing justice in specified cases by legislative and judicial action and by compelling men to work in the open—not some inclusive scheme of social reorganisation."

